# Supplementary material for: A Diamond/Graphene/Diamond Electrode for Waste Water Treatment
Source: Nanomaterials (Basel). 2023 Nov 29;13(23):3043. doi: 10.3390/nano13233043 (PMC10707964; doi:10.3390/nano13233043)
Supplement: Supplementary file 1 [file nanomaterials-13-03043-s001.zip › nanomaterials-2679679-supplementary.pdf]

Supplementary Materials

A Diamond/Graphene/Diamond Electrode for Waste Water Treatment

Yibao Wang [1], Zhigang Gai [1,*], Fengxiang Guo [1], Mei Zhang [1], Lili Zhang [1], Guangsen Xia [1], Xu Chai [1], Ying Ren [2], Xueyu Zhang [1,*] and Xin Jiang [3]

Table. S1 The detailed preparation parameters of DGD films

**The preparation steps**	**Process**	**Gas atmosphere**	**Temperature**	**Time**
**First layer deposition of BDD**	Hot filament chemical vapor deposition	H_2: 980 sccm<br>CH_4: 10 sccm<br>4 kPa<br>$B(CH_3)_3$: 8 sccm	830-850 ℃	5 h
**Deposition of copper layer**	Electron beam evaporation	Vacuum: 2.3×10^{-3} Pa	/	1h
**Induced graphene layer**	Vacuum annealing	Vacuum: 0.01 Pa	1000℃	10min<br>20min<br>30min<br>40min
**Etched residual copper**	$CuSO_4$:5g<br>HCl: 25mL<br>H_2O:25mL	Atmosphere	Room temperature<br>27℃	2h
**Second layer deposition of BDD**	Hot filament chemical vapor deposition	H_2:980 sccm<br>CH_4: 10 sccm<br>4 kPa<br>$B(CH_3)_3$: 8 sccm	830-850 ℃	2h

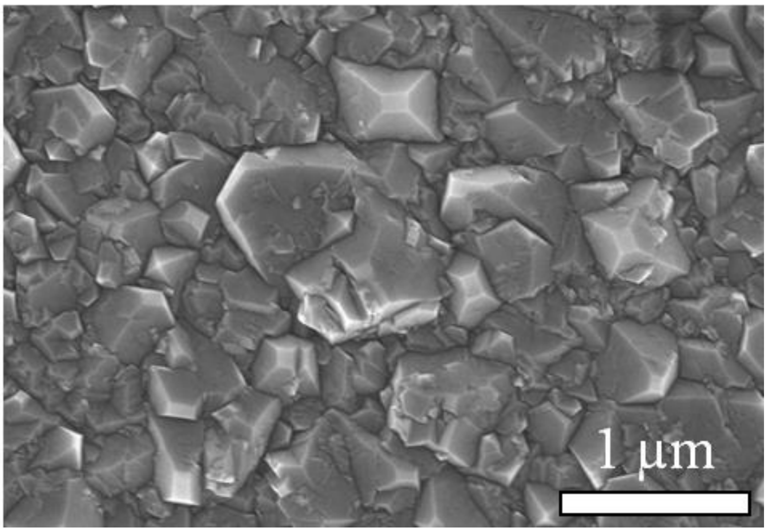

Figure S1. SEM images of DGD films after the growth of the upper BDD film

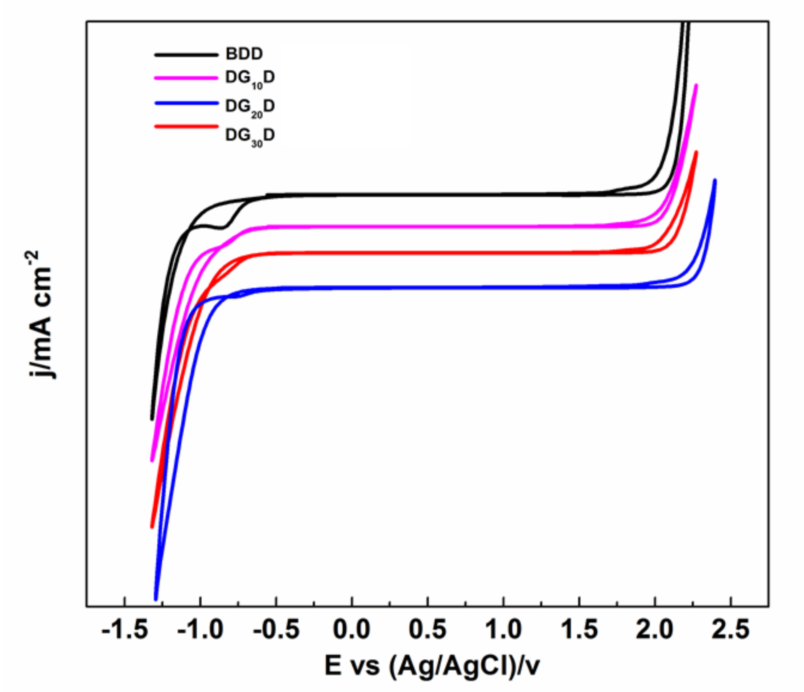

Figure S2. CV in 0.1 M H2SO4 solution of the electrode

Table S1 show the The detailed preparation parameters of DGD films . Figure S1 show the SEM images of GDG films after the growth of the upper BDD films. Upper films is very dense. Figure S2 show the CV in 0.1 M H2SO4 solution of the different electrods. We found that the oxygen evolution potential of each electrode was above 2.3v after the introduction of the interlayer, and the electrochemical windows was not narrowed.

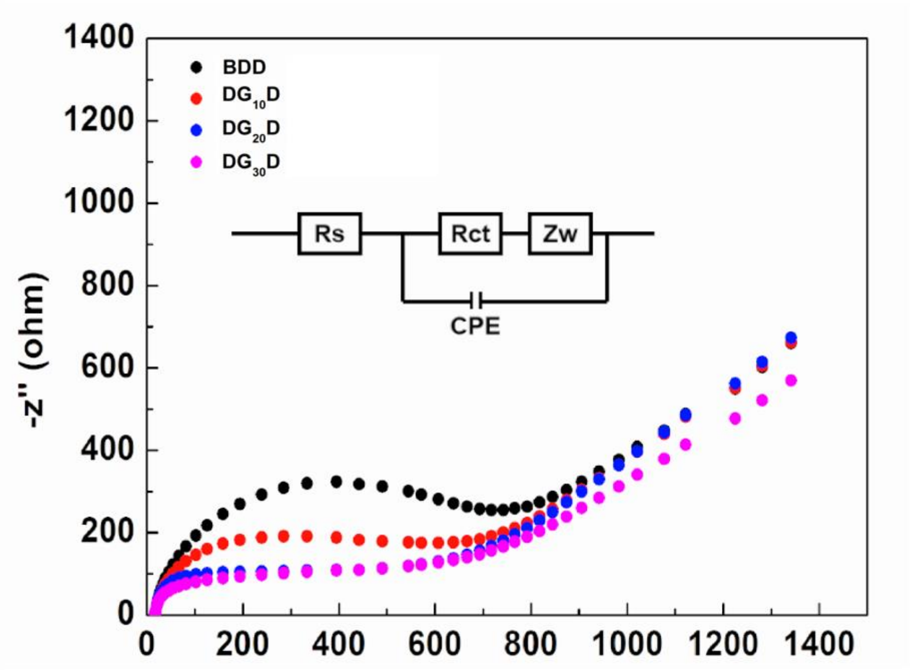

Figure S3 EIS in 0.1 M Na_2SO_4 solution containing 1 mM $[Fe(CN)_6]^{3-/4-}$, insert was the equivalent circuit

The Nyquist curve tested by EIS and Randles equivalent circuit are given in Figure S3. The Nyquist curve consists of an arc in the mid-high frequency regin and a sloping line in the low frequency region. The Randles equivalent circuit include the internal resistance (Rs) of electrochemical system, and charge transfer resistance (Rct) at the electrode/solution interface ,the Warburg impedance (Zw) of the substance diffusion and the constant phase element (CPE) of the double layer capacitance. Rct reflects the electron transfer capability of electrode at liquid-solid interface. The Rct values of BDD, $BG_{10}D$, $BG_{20}D$ and $BG_{30}D$ electrodes are 652, 447, 245 , 260 Ω, as the intermediate layer thickens, the BDD-30min-BDD Rct only 37% of that BDD. The BDD/G/BDD structure creates a low Rct,and the enhanced charge transfer at the liquid-solid interface can facilitate the direct oxidation reaction of environmental organic pollutants.

Table S2 main and by-products produced during the electrolysis of three organic matter

**Organic Matter**	**Molecular Formula**	**Decomposition process**	**The end product**	**By product**
Catechol	$C_6H_6O_2$	$BDD+H_2O \rightarrow BDD(HO^{\cdot})_{ads}+H^++e^-$	CO_2 ,H_2O	NO
Citric Acid	$C_6H_8O_7$	$BDD(HO^{\cdot})_{ads}+R \rightarrow BDD+CO_2+H_2O+e^-$	CO_2 ,H_2O	NO

		BDD(HO$^{\cdot}$)$_{ads}$→BDD+$\frac{1}{2}O_2$+H^++e^-		
TCH	$C_{22}H_{25}ClN_2$ 0_8	BDD+H_2O→BDD(HO$^{\cdot}$)$_{ads}$+H^++e^-<br>BDD(HO$^{\cdot}$)$_{ads}$+R→BDD+ Intermediate Products+H_2O+e^-<br>… (Multiple steps) …<br>BDD(HO$^{\cdot}$)$_{ads}$+M →BDD+CO_2+H_2O+e^-	CO_2,H_2O	10 Intermediate Products
